# Supplementary material for: Activation of AMPK/miR-181b Axis Alleviates Endothelial Dysfunction and Vascular Inflammation in Diabetic Mice
Source: Antioxidants (Basel). 2022 Jun 9;11(6):1137. doi: 10.3390/antiox11061137 (PMC9220246; doi:10.3390/antiox11061137)
Supplement: Supplementary file 1 [file antioxidants-11-01137-s001.zip › antioxidants-1756129-supplementary.pdf]

*antioxidants*

MDPI

*Supplementary information*

# Activation of AMPK/miR-181b Axis Alleviates Endothelial Dysfunction and Vascular Inflammation in Diabetic Mice

**Chak Kwong Cheng [1,2,†], Wenbin Shang [2,†], Jian Liu [3], Wai San Cheang [4], Yu Wang [2], Li Xiang [2], Chi Wai Lau [2], Jiang-Yun Luo [2], Chi Fai Ng [5], Yu Huang [1,2,*] and Li Wang [1,2,*]**

[1] Department of Biomedical Sciences, City University of Hong Kong, Hong Kong 999077, China; superandy333@gmail.com (C.K.C.)
[2] School of Biomedical Sciences, The Chinese University of Hong Kong, Hong Kong 999077, China; superandy333@gmail.com (C.K.C.); wenbinmedical@gmail.com (W.S.); wangyu.hmu@gmail.com (Y.W.); xiangli@hkbu.edu.hk (L.X.); chiwailau@cuhk.edu.hk (C.W.L.); luojiangyun1985@gmail.com (J.-Y.L.)
[3] Department of Integration of Chinese and Western Medicine, School of Basic Medical Sciences, Peking University, Beijing 100871, China; liujianpku@bjmu.edu.cn (J.L.)
[4] State Key Laboratory of Quality Research in Chinese Medicine, Institute of Chinese Medical Sciences, University of Macau, Zhuhai 519000, China; annacheang@um.edu.mo (W.S.C.)
[5] Department of Surgery, The Chinese University of Hong Kong, Hong Kong 999077, China; ngcf@surgery.cuhk.edu.hk (C.F.N.)
* Correspondence: yu.huang@cityu.edu.hk; Tel.: +852-3442-7746 (Y.H.); li.wang@cityu.edu.hk; Tel.: +852-3442-9187 (L.W.)
† Chak Kwong Cheng and Wenbin Shang contribute equally to this study.

## Supplementary Materials

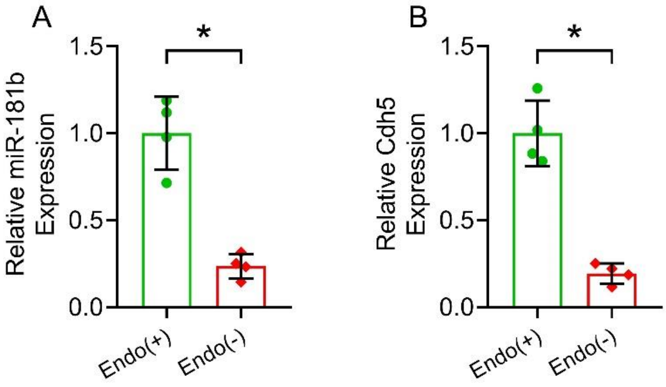


**Figure S1.** Expression of miR-181b and Cdh5 in aortas of C57BL/6 mice upon endothelium removal. Expression of **(A)** miR-181b and **(B)** endothelial cell-specific Cdh5 in mouse aortas. n=4 per group. Data are mean ± SD. *$p < 0.05$ vs Endo(+) (unpaired t-test and nonparametric Mann-Whitney test).

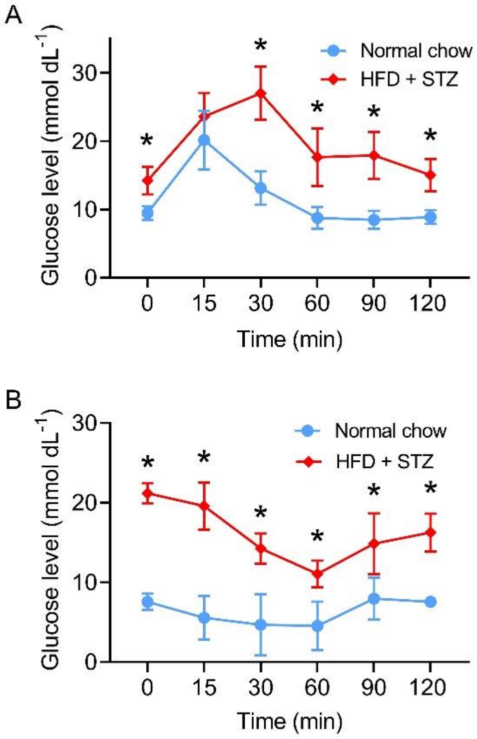


**Figure S2.** Glucose homeostasis of diabetic mice. **(A)** GTT, and **(B)** ITT of normal chow-fed mice and HFD/STZ-induced diabetic mice. n=10 per group. Data are mean ± SD. *$p < 0.05$ vs Normal chow (unpaired t-test and nonparametric Mann-Whitney test).
